# IMCA Induces Ferroptosis Mediated by SLC7A11 through the AMPK/mTOR Pathway in Colorectal Cancer

**DOI:** 10.1155/2020/1675613

**Published:** 2020-04-03

**Authors:** Lei Zhang, Wen Liu, Fangyan Liu, Qun Wang, Mengjiao Song, Qi Yu, Kun Tang, Tieshan Teng, Dongdong Wu, Xijing Wang, Wuqi Han, Yanzhang Li

**Affiliations:** ^1^Cell Signal Transduction Laboratory, Bioinformatics Center, Laboratory for Nanomedicine, Henan International Joint Laboratory for Nuclear Protein Regulation, School of Basic Medical Sciences, Henan University, Kaifeng 475004, China; ^2^Department of Dermatology, Second People's Hospital of Zhengzhou, Zhengzhou 450006, China; ^3^Kaifeng Food and Drug Inspection Institute, Kaifeng 475004, China

## Abstract

Ferroptosis, implicated in several diseases, is a new form of programmed and nonapoptotic cell death triggered by iron-dependent lipid peroxidation after inactivation of the cystine/glutamate antiporter system xc^–^, which is composed of solute carrier family 7 membrane 11 (SLC7A11) and solute carrier family 3 membrane 2 (SLC3A2). Therefore, inducing ferroptosis through inhibiting the cystine/glutamate antiporter system xc^–^ may be an effective way to treat cancer. In previous screening tests, we found that the benzopyran derivative 2-imino-6-methoxy-2H-chromene-3-carbothioamide (IMCA) significantly inhibited the viability of colorectal cancer cells. However, the impact of IMCA on ferroptosis remains unknown. Hence, this study investigated the effect of IMCA on ferroptosis and elucidated the underlying molecular mechanism. Results showed that IMCA significantly inhibited the cell viability of colorectal cancer cells *in vitro* and inhibited tumor growth with negligible organ toxicity *in vivo*. Further studies showed that IMCA significantly induced the ferroptosis of colorectal cancer cells. Mechanistically, IMCA downregulated the expression of SLC7A11 and decreased the contents of cysteine and glutathione, which resulted in reactive oxygen species accumulation and ferroptosis. Furthermore, overexpression of SLC7A11 significantly attenuated the ferroptosis caused by IMCA. In addition, IMCA regulated the activity of the AMPK/mTOR/p70S6k signaling pathway, which is related to the activity of SLC7A11 and ferroptosis. Collectively, our research provided experimental evidences on the activity and mechanism of ferroptosis induced by IMCA and revealed that IMCA might be a promising therapeutic drug for colorectal cancer.

## 1. Introduction

Colorectal cancer (CRC) is a common malignant tumor and an important health problem worldwide. According to the cancer statistics worldwide for 36 cancers in 185 countries in 2018, both sexes combined, colorectal cancer is the third diagnosed cancer (10.2% of the total cases) globally and the second leading cause (10.2% of total cancer deaths) of cancer-related deaths worldwide [1, 2]. Conventional treatment options for cancer include chemotherapy, radiation, and surgery [3]. In addition, new treatment methods, such as biotargeted therapy, immunotherapy, and precise treatment, have been gradually applied for CRC treatment [4, 5]. In order to improve the therapeutic effect, chemotherapy, radiation, and surgery are often used in combination. However, conventional treatments are often associated with serious side effects and toxicity, thus significantly affecting patients' quality of life. In addition, cancer cells have also been found to be able to develop resistance toward chemotherapy and radiotherapy over time. Therefore, it is still an important task for researchers to find new drugs with high efficiency and low side effects for CRC.

The mainly regulated cell deaths are apoptosis, necroptosis, autophagy, ferroptosis, and pyroptosis, which are believed to be critical for development, homeostasis, disease occurrence, and treatment, such as malignant tumors [6–8]. Identified as a new mode of programmed cell death in 2012, ferroptosis is a unique iron-reliant and reactive oxygen species- (ROS-) dependent form of nonautophagic and nonapoptotic programmed cell death [6, 9]. Mitochondrial morphological change is characterized by decreased or vanished mitochondria cristae, a ruptured outer mitochondrial membrane, and a condensed mitochondrial membrane in ferroptotic cells [7, 9]. The small molecule erastin induces ferroptosis through inhibiting the import of cystine, which is decomposed into two molecules of cysteine (Cys), resulting in glutathione (GSH) exhaustion and inactivation of the phospholipid peroxidase glutathione peroxidase 4 (GPX4) [8, 10]. GPX4 reduces the toxic lipid hydroperoxides (L-OOH) to the nontoxic lipid alcohols (L-OH) by oxidizing GSH to glutathione disulfide (GSSG) [8, 11]. Cystine, one of the raw materials for the synthesis of the major antioxidant GSH, is transported to the intracellular space through transporting glutamate to the extracellular space by a heterodimeric cystine/glutamate antiporter system xc^−^, which is mainly composed of a twelve-pass transmembrane catalytic subunit solute carrier family 7 member 11 (SLC7A11) and a single-pass transmembrane anchoring protein solute carrier family 3 member 2 (SLC3A2) [7, 12]. Pharmacological inhibition of the system xc^−^-reliant antioxidant defense system leads to ROS accumulation and ferroptosis, such as erastin, sulfasalazine, and sorafenib [7].

2-Imino-6-methoxy-2H-chromene-3-carbothioamide (IMCA) is a benzopyran derivative, with a wide variety of biological activities for the treatment of cancer, type 2 diabetes, inflammation, skin diseases, Alzheimer's disease (AD), the polycystic kidney disease, and viral and bacterial infections [13]. We report for the first time that IMCA inhibits the viability of medullary thyroid cancer through inducing apoptosis [14]. In the course of studying the anti-CRC effect of IMCA, we found for the first time that IMCA leads to the death of CRC cells.

In the current study, two types of CRC cell lines and xenograft model were utilized to evaluate the anti-CRC effects and the mechanism of IMCA. The effects of IMCA on the biological phenotype of CRC cells were examined, and the underlying molecular mechanisms of IMCA-induced ferroptosis were elucidated. Mechanistically, we found that IMCA downregulated the expression of SLC7A11 and decreased the contents of Cys and glutathione, which resulted in ROS accumulation and ferroptosis. Furthermore, overexpression of SLC7A11 significantly attenuated ferroptosis caused by IMCA through downregulating the expression of SLC7A11. In addition, IMCA regulated the activity of the AMPK/mTOR/p70S6k signaling pathway, which is related to the activity of SLC7A11 and ferroptosis. For the first time, we found a novel small-molecule compound against CRC and demonstrated that IMCA induced ferroptosis mediated by SLC7A11 through the AMPK/mTOR pathway in CRC.

## 2. Materials and Methods

### 2.1. Cell Lines and Cell Culture

Human CRC cell lines DLD-1 and HCT-116 were purchased from a typical cell culture collection committee of the Chinese Academy of Sciences Library (Shanghai, China). These cells were grown in Roswell Park Memorial Institute 1640 (Gibco, 11875119) medium containing 10% heat-inactivated fetal bovine serum (Gibco, 16000-044), 100 U/mL penicillin and 100 *μ*g/mL streptomycin (Solarbio, P1400), and 5% CO_2_ at 37°C. The solvent dimethyl sulfoxide (DMSO) used in the experiments was less than 0.1%.

### 2.2. Chemicals and Reagents

IMCA was purchased from Tao Su Biochemical Technology Co. Ltd. (AE-848/32005043, Shanghai, China). GPX4, glutathione synthetase (GSS), and SLC7A11 antibodies were purchased from Proteintech Co. Ltd. (Wuhan, China). PCR primers were designed and synthesized by Sangon Biotech Co. Ltd. (Shanghai, China). The SYBR green PCR Master Mix was purchased from Thermo Scientific (Waltham, MA, USA). MTT cell proliferation and cytotoxicity detection kit, ROS detection kit, GSH detection kit, GSSG detection kit, and Cys detection kit were purchased from Solarbio Co. Ltd. (Beijing, China).

### 2.3. Cell Viability Assay

IMCA cytotoxicity was detected with the MTT cell proliferation and cytotoxicity detection kit in accordance with the manufacturer's instructions. In brief, DLD-1 and HCT-116 cells were seeded into 96-well cell culture plates with 5 × 10^3^ cells per well and cultured continuously for 12 h. Different intervention reagents were added to the cultural plates and cultured continuously for 48 h. A 10 *μ*L MTT solution (10 mg/mL in PBS) was added to the cultural plates and cultured continuously for 4 h. Then, 100 *μ*L of DMSO was added to the cultural plates and the absorbance of the samples was measured using a multifunctional enzyme marker (Varioskan Flash, Thermo Scientific) at the wave length of 570 nm.

### 2.4. ROS Analysis

ROS was detected with the ROS detection kit in accordance with the manufacturer's instructions. Briefly, DLD-1 and HCT-116 cells were seeded into 6-well cell culture plates with 2 × 10^5^ cells per well and cultured continuously for 12 h. Different intervention reagents were added to the cultural plates and cultured continuously for 48 h.

#### 2.4.1. Detection with Multifunctional Enzyme Marker

Cells were harvested and washed once with PBS. The harvested cells were suspended in DCFH-DA, diluted 1000 times in serum-free medium, and incubated for 20 min. Subsequently, the cells were washed three times with serum-free medium and were then detected with a multifunctional enzyme marker (Varioskan Flash, Thermo Scientific) at the excitation wavelength of 488 nm and emission wavelength of 525 nm.

#### 2.4.2. Detection by Confocal Microscopy

As previously described [15], the cells were washed once with PBS, incubated in DCFH-DA, and diluted 1000 times in serum-free medium for 20 min. Then, the cells were washed three times with serum-free medium and detected via confocal microscopy (A1R+Storm, Nikon).

### 2.5. Cys Analysis

Cys was detected with the Cys detection kit according to the manufacturer's instructions. Briefly, DLD-1 and HCT-116 cells were seeded into 6-well cell culture plates with 2 × 10^5^ cells per well and cultured continuously for 12 h. Different intervention reagents were added to the cultural plates and cultured continuously for 48 h. The cells were crushed by ultrasound and centrifuged at 8000 g for 10 min. Then, 100 *μ*L of the supernatant, 500 *μ*L of reagent I, and 500 *μ*L of reagent II were mixed and detected with a multifunctional enzyme marker (Varioskan Flash, Thermo Scientific) at the wavelength of 600 nm.

### 2.6. GSH Analysis

GSH was detected with the GSH detection kit in accordance with the manufacturer's instructions. Briefly, DLD-1 and HCT-116 cells were seeded into 6-well cell culture plates with 2 × 10^5^ cells per well and cultured continuously for 12 h. Different intervention reagents were added to the cultural plates and cultured continuously for 48 h. Cells were harvested and washed twice with PBS. The cells were resuspended in reagent I and frozen and thawed three times with liquid nitrogen. A 20 *μ*L supernatant from the cell suspension centrifuged at 8000 rpm was mixed with 140 *μ*L of reagent II and 40 *μ*L of reagent III and detected with a multifunctional enzyme marker (Varioskan Flash, Thermo Scientific) at the wavelength of 412 nm.

### 2.7. qRT-PCR Assay

As previously described [16], total RNA was extracted using Trizol reagent in accordance with the manufacturer's instructions. mRNA was reversed transcribed into cDNA with the PrimeScript RT reagent kit (Takara, DRR047A) in a 20 *μ*L reaction system. qPCR analysis was conducted with on an ABI 7500 Fast Real-Time PCR System (Applied Biosystems, Waltham, MA, USA). The PCR primers are listed in Table 1.

### 2.8. Western Blot Analysis

As previously described [17], protein expression was determined by Western blot in accordance with standard protocols. Briefly, cells were harvested and lysed in RIPA buffer with a protease inhibitor. Cell lysates were quantitated with a Bradford reagent, separated with a denatured sodium dodecyl sulfate 4%-20% polyacrylamide gel electrophoresis (SDS-PAGE), and transferred onto a polyvinylidene fluoride (PVDF) membrane through wet electroblotting. The PVDF membrane was blocked with dried skimmed milk and incubated with primary antibodies specific for GPX4, GSS, and SLC7A11. A HRP-conjugated secondary antibody was incubated at room temperature for 2 h, and the blot analysis was visualized with a chemiluminescence analyzer (Amersham Biosciences, Boston, MA, USA).

### 2.9. Overexpression of SLC7A11

The recombinant overexpression plasmid of SLC7A11 was constructed by Hanbio Technical Co., Ltd. (Shanghai, China). The overexpression plasmid of SLC7A11 was transfected into DLD-1 and HCT-116 cells by Lipofectamine 3000 and P3000 to produce SLC7A11-overexpressing transient cell lines. After 48 h of transfection, the cells were collected and transfection efficiency was determined using Western blot.

### 2.10. In Vivo Experiments

As previously described [18], the animal experiments involved in this project have been approved by the Medical and Scientific Research Ethics Committee of Henan University School of Basic Medical Sciences. Five-week-old female BALB/c nude mice were purchased from Beijing Weitong Lihua Experimental Animal Technical Co., Ltd. (Beijing, China). 10^6^ DLD-1 cells suspended in normal saline were injected subcutaneously into nude mice. The volume of tumors was calculated using the following formula: L (the long diameter) × W (the short diameter) × W × 1/2. The mice were randomly assigned to the treatment and control groups until the tumor size reached approximately 100 mm^3^. The mice in the treatment group were injected with 0.174 mg/mL IMCA (100 *μ*L), and those in the control group were injected with an equal volume of normal saline. The nude mice were euthanized, and samples were obtained from their tumor, heart, hepar, kidney, and blood after 33 days of IMCA treatment. The serum was separated, and alanine aminotransferase and urea nitrogen were determined using the animal specific automatic biochemical analyzer (Catalyst Dx, IDEXX, Maine, USA) to evaluate the effects of IMCA on liver and kidney functions. Blood was obtained, and the number of blood cells was determined by an animal hematology analyzer (BC-5000 vet, Mindray, Shenzhen, China) to evaluate the effect of IMCA on blood routine. Organ index was calculated by dividing organ mass by body weight and multiplying by 100% to evaluate the effect of IMCA on organs.

### 2.11. Transmission Electron Microscopy

DLD-1 and HCT-116 cells were seeded into 6-well cell culture plates with 2 × 10^5^ cells per well and cultured continuously for 12 h. Different intervention reagents were added to the cultural plates and cultured continuously for 48 h. Cells were harvested and fixed in 2% glutaraldehyde. Samples were treated and detected in the electron microscopy room of Xi'an Jiaotong University.

### 2.12. Statistical Analysis

All statistical analyses were completed by SPSS16.0. The statistical difference between the treatment and control groups of IMCA was analyzed by Student's *t*-test.

## 3. Results

### 3.1. IMCA Reduced the Viability of CRC Cell Lines

**Figure 1:**
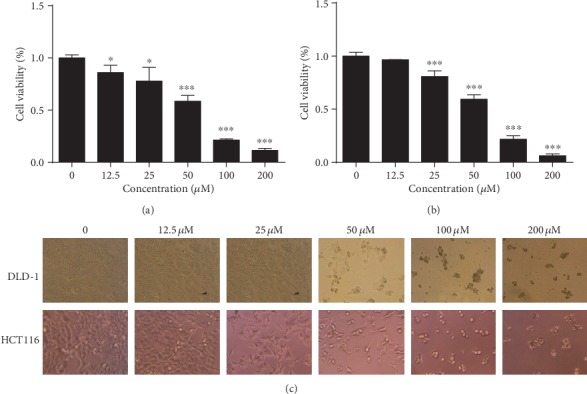
IMCA reduced the viability of CRC cell lines. Human CRC cell lines DLD-1 and HCT-116 were treated with different concentrations of IMCA (12.5-200 *μ*M) for 48 h. The cell viability was determined by the MTT assay kit at 48 h. Results showed that IMCA significantly reduced the viability of DLD-1 (a) and HCT116 (b) in a dose-dependent manner *in vitro*. The values of viability were expressed as mean ± standard deviation. (c) The cell morphology treated with different concentrations of IMCA (12.5-200 *μ*M) for 48 h was photographed under an inverted microscope. ^∗^*p* < 0.05; ^∗∗∗^*p* < 0.001.

The cell viability of human CRC cell lines DLD-1 and HCT116 treated with different concentrations of IMCA for 48 h was determined using the MTT method to confirm the inhibition efficiency of IMCA on the proliferation of CRC cell lines. The viability of the two CRC cell lines significantly reduced in a dose-dependent manner compared with that of the control cells (Figures 1(a) and 1(b)). The IC50 values of IMCA for DLD-1 and HCT116 cells were 50.2 *μ*M and 44.5 *μ*M, respectively. The cell morphology was photographed with an inverted microscope (Figure 1(c)). Compared with the control group cells, the CRC cells treated with IMCA for 48 h were characterized by shattered, metamorphous, and multidirectional cell morphology. The above results showed that IMCA significantly reduced the viability of CRC cell *in vitro*.

### 3.2. IMCA Inhibited the Growth of Xenograft In Vivo

**Figure 2:**
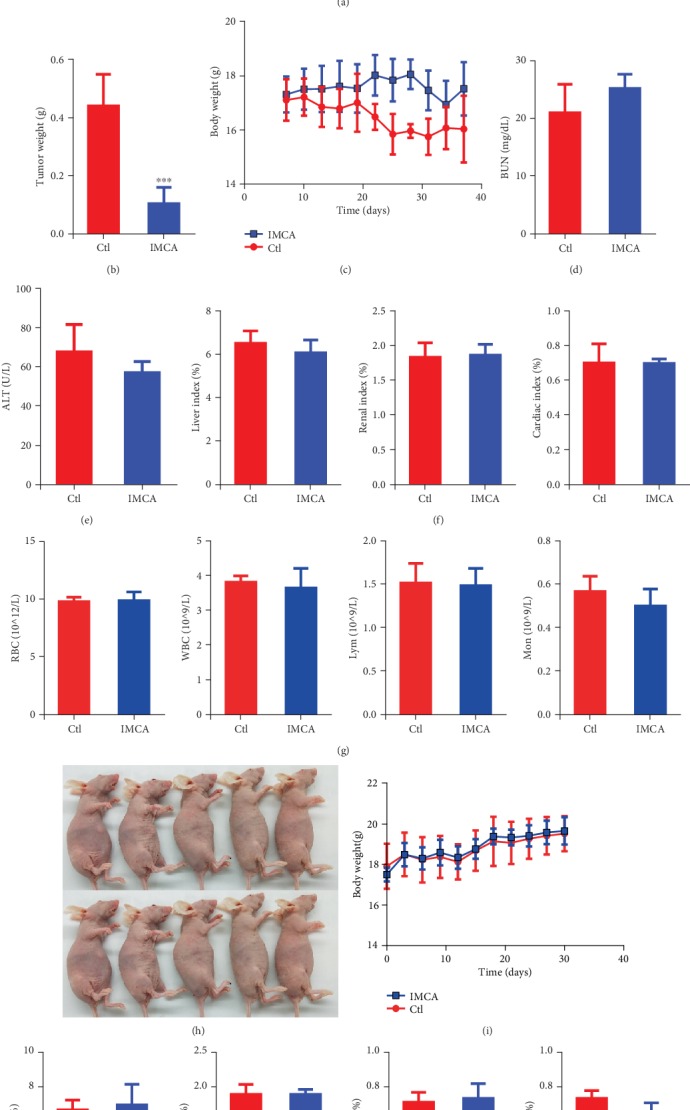
IMCA inhibited the growth of xenograft *in vivo*. (a) Representative photographs of tumor-bearing nude mice and tumor volume changes of mice in the experimental period. (b) Tumor weight was determined and compared between the IMCA treatment and control groups. (c) Body weight changes of mice in the experimental period. BUN (d) and ALT (e) were determined and compared between the IMCA treatment and control groups. (f) Liver, renal, and cardiac indices were determined and compared between IMCA treatment groups and controls. (g) RBC, WBC, Lym, and Mon contents were determined and compared between IMCA treatment and control groups. (h) Representative photographs of nontumor-bearing nude mice. (i) Body weight changes of nontumor-bearing nude mice in the experimental period. (j) Liver, renal, cardiac, and spleen indices were determined and compared between the IMCA treatment and control groups. ^∗∗^*p* < 0.01; ^∗∗∗^*p* < 0.001.

Since we observed a significantly inhibitory effect of IMCA on CRC cell viability, we next dissected the antitumor effects of IMCA using the BALB/c nude mouse xenografts bearing DLD-1 cells *in vivo*. As shown in Figures 2(a) and 2(b), the tumor volume and weight in the IMCA-treated group mice dramatically reduced and the inhibition rate reached 76.4% compared with those in the saline negative control group. We monitored the body weight of nude mice every three days during the treatment period to determine the impact of IMCA on the health of the mice. The nude mice did not significantly lose weight during the entire treatment period compared with the control group (Figure 2(c)). For health measurements, we also measured liver and kidney function index, organ index, and blood routine, including alanine aminotransferase (ALT), blood urea nitrogen (BUN), heart index, liver index, kidney index, red blood cells (RBC), white blood cells (WBC), lymphocytes (Lym), and monocytes (Mon) (Figures 2(d)–2(g)). The results showed that no significant health figure changes were observed. To further assess the toxicity of IMCA on healthy animals, nontumor-bearing nude mice were injected with equal doses of IMCA and normal saline through the tail vein, and the body weight of nude mice was monitored every three days during the treatment period. Results showed that IMCA did not significantly affect the weight gain of nude mice, compared with the normal saline groups (Figures 2(h) and 2(i)). There were no significant changes in liver, kidney, heart, and spleen indices, compared with the normal saline groups (Figure 2(j)). Collectively, the above data indicate that IMCA significantly inhibits tumor growth with negligible organ toxicity *in vivo*.

### 3.3. IMCA Induced ROS-Mediated Ferroptosis of CRC Cell Lines

**Figure 3:**
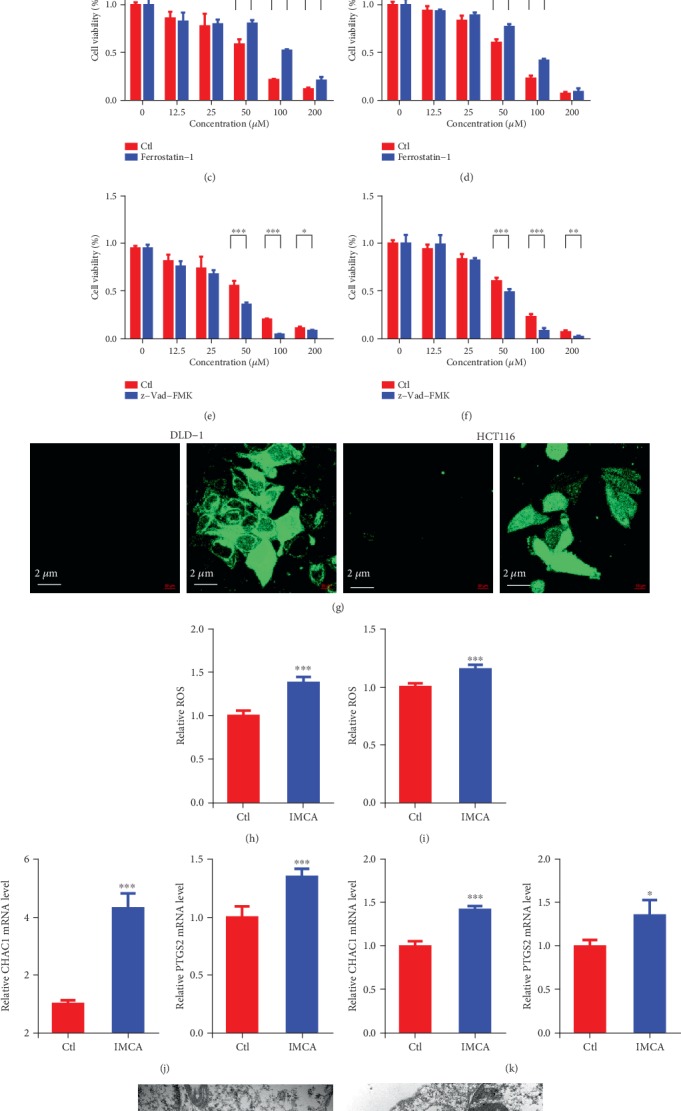
IMCA induced the ROS-mediated ferroptosis of CRC cell lines *in vitro*. Human CRC cell lines DLD-1 and HCT-116 were treated with different concentrations of IMCA (12.5–200 *μ*M) with or without DFO, Ferrostatin-1, and z-Vad-FMK for 48 h. The cell viability was determined by the MTT assay kit at 48 h. Results showed that iron chelator DFO (0.2 *μ*M) rescued the cell viability inhibited by IMCA in DLD-1 (a) and HCT116 (b) cell lines. The cell viability was also rescued by ferroptosis inhibitor Ferrostatin-1 (2 *μ*M) in DLD-1 (c) and HCT116 (d) cell lines. The results also showed that apoptosis inhibitor z-Vad-FMK (3 *μ*M) did not rescue the cell viability inhibited by IMCA in DLD-1 (e) and HCT116 (f) cell lines. (g) Confocal laser scanning microscope images of ROS generation were obtained and compared between the IMCA treatment and control groups in DLD-1 and HCT116 cells. Relative ROS accumulation was determined with a multifunctional enzyme marker in DLD-1 (h) and HCT116 (i) cells. Relative mRNA expression of ferroptosis markers CHAC1 and PTGS2 was determined and compared between the IMCA treatment and control groups in DLD-1 (j) and HCT116 (k) cells. Transmission electron microscopy images of mitochondrial morphology were obtained and compared between the IMCA treatment groups (m) and control groups (l) in DLD-1 cells. The black tip points to mitochondria. ^∗^*p* < 0.05; ^∗∗^*p* < 0.01; ^∗∗∗^*p* < 0.001.

Ferroptosis is a unique iron-reliant and nonapoptotic form of programmed cell death, which is characterized by ROS accumulation induced by lipid peroxidation and ineffective GPX4 [19, 20]. In order to determine the cause that cell viability was inhibited by IMCA, human CRC cell lines DLD-1 and HCT-116 were treated with different concentrations of IMCA (12.5–200 *μ*M) with or without iron chelator DFO, ferroptosis inhibitor Ferrostatin-1, or apoptosis inhibitor z-Vad-FMK for 48 h. Results showed that both DFO and Ferrostatin-1 rescued the cell viability induced by IMCA, while z-Vad-FMK failed to rescue the cell viability induced by IMCA (Figures 3(a)–3(f)). To further establish the impact of IMCA on ferroptosis, we next sought to determine the ROS accumulation induced by IMCA using a confocal microscope and a multifunctional microplate reader. Results showed that IMCA significantly induced ROS accumulation at a concentration of 50 *μ*M for 48 h in DLD-1 and HCT116 cells (Figures 3(g)–3(i)). The morphological changes of ferroptosis were mainly characterized by membrane thickening, mites disappearing, and rupture of the mitochondria [6]. To establish the impact of IMCA on mitochondrial morphology, we next examined the mitochondrial morphology of the cells treated with IMCA under a transmission electron microscope. Results showed that IMCA significantly induced the disappearance of mitochondrial crista at a concentration of 50 *μ*M in DLD-1 cells (Figures 3(l)–3(m)). Gene expression markers associated with cells undergoing ferroptosis include increases in CHAC1 and PTGS2 mRNA expression [21–23]. IMCA significantly induced the mRNA expression of CHAC1 and PTGS2 at a concentration of 50 *μ*M in DLD-1 and HCT116 cells (Figures 3(j)–3(k)). Collectively, these data suggest that IMCA induces CRC cell ferroptosis *in vitro*.

### 3.4. IMCA Inhibited the Expression of SLC7A11 In Vitro

**Figure 4:**
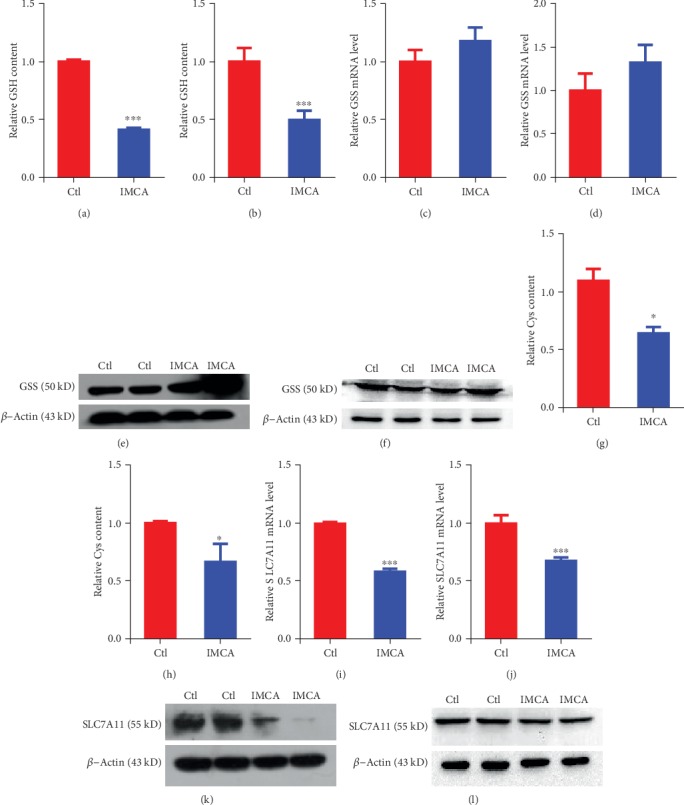
IMCA inhibited the expression of SLC7A11. The relative GSH contents were determined and compared between the IMCA treatment and control groups in DLD-1 (a) and HCT116 (b) cells. Relative mRNA expression of GSS was determined and compared between the IMCA treatment and control groups in DLD-1 (c) and HCT116 (d) cells. Relative protein expression of GSS was determined and compared between the IMCA treatment and control groups in DLD-1 (e) and HCT116 (f) cells. The relative Cys contents were determined and compared between the IMCA treatment and control groups in DLD-1 (g) and HCT116 (h) cells. Relative mRNA expression of SLC7A11 was determined and compared between the IMCA treatment and control groups in DLD-1 (i) and HCT116 (j) cells. Relative protein expression of SLC7A11 was determined and compared between the IMCA treatment and control groups in DLD-1 (k) and HCT116 (l) cells. ^∗^*p* < 0.05; ^∗∗^*p* < 0.01; ^∗∗∗^*p* < 0.001.

Ferroptosis is characterized by the accumulation of ROS, which is scavenged by GPX4 through conversion of reduced GSH into the oxidized form GSSG [24–26]. Therefore, the expression of GPX4 and the GSH level were explored and we found that IMCA significantly reduced GSH levels with negligible impact on the expression of GPX4 in DLD-1 and HCT116 cells (Figures 4(a) and 4(b); Fig. [Supplementary-material supplementary-material-1]). GSH is synthesized from glutamate, Cys, and glycine by the ATP-dependent catalysis of glutathione synthetase (GSS) [27]. The rate of GSH synthesis is primarily limited by the Cys content [28]. The expression of GSS and the Cys level were determined to elucidate the mechanism of GSH reduction triggered by IMCA. Results showed that IMCA significantly reduced Cys levels with negligible impact on the expression of GSS in DLD-1 and HCT116 cells with negligible changes in the expression of GSS (Figures 4(c)–4(h)). The heterodimeric cystine/glutamate antiporter system xc^−^ transports Cys into the intracellular space to synthesize GSH, which inhibited ferroptosis. SLC7A11 is the catalytic subunit of system xc^−^ [29]. The expression of SLC7A11was determined to dissect the mechanism by which IMCA triggers Cys reduction. Results showed that IMCA significantly reduced the expression of SLC7A11 in DLD-1 and HCT116 cells (Figures 4(i)–4(m)). Collectively, these data suggest that IMCA induces CRC cell ROS accumulation and ferroptosis by downregulating SLC7A11 expression, inhibiting Cys transport and reducing GSH synthesis *in vitro*.

### 3.5. Overexpression of SLC7A11 Rescues IMCA-Induced Ferroptosis of CRC Cells In Vitro

**Figure 5:**
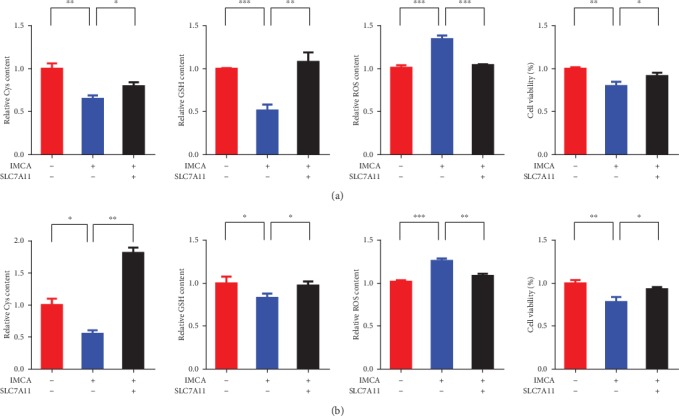
The overexpression of SLC7A11 rescues IMCA-induced CRC cell ferroptosis *in vitro*. Overexpression of SLC7A11 restored the decrease of Cys and glutathione content, the increase of ROS, and the decrease of cell viability induced by IMCA in DLD-1 (a) and HCT116 (b) cells.

SLC7A11 plays an important role in regulating ROS-mediated ferroptosis. Knocking down the expression of SLC7A11 results in elevated levels of endogenous ROS levels. Overexpression of SLC7A11 results in a cancer stem cell phenotype that contributes to severe chemoresistance [30, 31]. *SLC7A11*-overexpressing DLD-1 and HCT116 CRC cells were generated. Cys, GSH, ROS, and cell viability were detected, and results showed that the overexpression of SLC7A11 significantly rescued the IMCA-induced reduction of Cys, GSH, and cell viability and increased the ROS levels in *SLC7A11*-overexpressing DLD-1 and HCT116 CRC cells (Figures 5(a) and 5(b)). Collectively, these data suggest that *SLC7A11* inhibits the ferroptosis induced by IMCA.

### 3.6. IMCA Inhibits mTOR/P70S6K Activity through Phosphorylating AMPK

**Figure 6:**
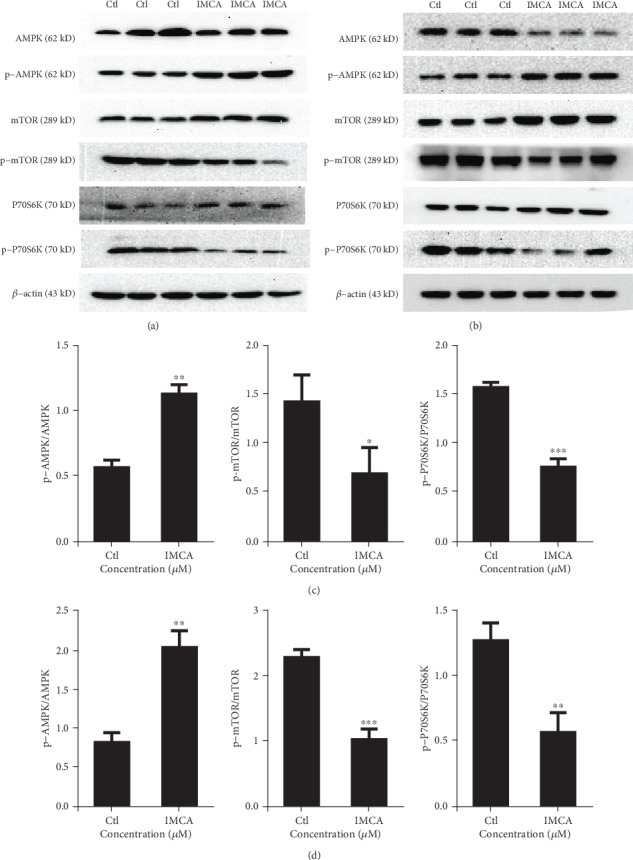
IMCA inhibited the mTOR/P70S6K activity through phosphorylating AMPK. DLD-1 (a) and HCT116 (b) cells were treated with 50 *μ*M IMCA for 48 h, and the protein expression was assessed by Western blot. The intensities of the p-AMPK, p-mTOR, and p-P70S6K bands were quantified by densitometry analyses and normalized by the amount of AMPK, mTOR, and P70S6K in DLD-1 (c) and HCT116 (d) cells, respectively (*n* = 3). ^∗^*p* < 0.05, ^∗∗^*p* < 0.01, and ^∗∗∗^*p* < 0.001 compared with the control group.

AMPK phosphorylation at Thr172 blocks the activity of SLC7A11, which inhibits the activity of system xc^−^ to transport cystine into cells and eventually leads to ferroptosis [12, 32]. As expected, the present studies showed that IMCA promoted the phosphorylation of AMPK in DLD-1 and HCT116 cells (Figures 6(a) and 6(b)). AMPK activation inhibits mTOR activity, which counteracts the elevated expression of SLC7A11 induced by APR246 and the protective cellular responses, and eventually results in cell death [33–35]. Furthermore, our results showed that the phosphorylation of mTOR and the downstream target protein P70S6K had been significantly decreased by 50 *μ*M IMCA treatment in DLD-1 and HCT116 cells.

## 4. Discussion

Chemotherapy is increasingly used in CRC as a complementary treatment strategy for CRC after surgery [36, 37]. In consideration of the high morbidity and mortality of CRC [2], new therapeutic drugs with high efficiency and low side effects for CRC must be developed. The present study showed that IMCA significantly inhibited the viability of human CRC cell lines DLD-1 and HCT116 (Figure 1). Further *in vivo* experiments showed that IMCA significantly inhibited the growth of xenograft and did not significantly affect the main organ index and blood biochemical parameters, such as aspartate transaminase (AST) and urea nitrogen (BUN). *In vitro* and *in vivo* results revealed that IMCA may be an effective drug candidate for CRC.

IMCA is a benzopyran derivative, provided with a wide variety of biological activities, including regulating cell death by ferroptosis execution [38]. For example, benzopyran derivative vitamin E hydroquinone is an endogenous regulator of ferroptosis [38]. Further transcript profile analysis showed that IMCA-regulated CRC cell death was associated with ferroptosis-related gene expression. Ferroptosis is a new form of nonautophagic and nonapoptotic programmed cell death characterized by the accumulation of lethal ROS and decreased or vanished mitochondria cristae [6, 10, 39]. Our results were consistent with the characteristics of ferroptosis, which showed that IMCA at 50 *μ*M significantly promoted the ROS accumulation and induced the disappearance of mitochondria in DLD-1 and HCT116 cells. Increased mRNA expression of CHAC1 and PTGS2 is considered a marker of ferroptosis cell death [8, 40]. Consistent with the characteristics of ferroptosis, our results showed that IMCA at 50 *μ*M significantly promoted the mRNA expression of CHAC1 and PTGS2 in DLD-1 and HCT116 cells. Overall, our results provided evidence that IMCA causes cell death through ferroptosis.

GSH is a momentous intracellular antioxidant that acts as a reducing substrate of GPX4 to mitigate the accumulation of ROS and protect cells from oxidative damage [7, 41]. Results showed that IMCA at 50 *μ*M significantly reduced the content of GSH in DLD-1 and HCT116 cells. One of the committed substrates used to synthesize GSH catalyzed by GSS is Cys, which has limited intracellular content owing to neurotoxicity [42–44]. Cys should be supplemented extracellularly to ensure that sufficient GSH is synthesized to protect cells from oxidative damage. Cys is derived from the decomposition of cystine, which is imported into the cells through the cystine/glutamate antiporter system xc^−^ [45, 46]. Inhibition of system xc^−^ significantly depletes the intracellular Cys, retards GSH synthesis, and eventually results in ferroptosis [47]. Erastin [6], sulfasalazine [48], sorafenib [6], Artesunate [49], Lanperisone [50], and Piperazine erastin [10] are the most investigated inhibitors targeting system xc^−^ and inducing ferroptosis. In the present study, IMCA did not significantly downregulate GSS expression but depleted the intracellular Cys. These results revealed that IMCA depleted the intracellular Cys and GSH and induced ferroptosis. We overexpressed SLC7A11 in DLD-1 and HCT116 cell lines to determine the role of SLC7A11 in ferroptosis induced by IMCA. We next determined the effect of SLC7A11 overexpression on cell Cys, GSH, and ROS amounts and cell viability regulated by IMCA in DLD-1 and HCT116 cells. As a result, overexpression of SLC7A11 recovered the Cys and GSH depleted by IMCA and inhibited the ROS levels enhanced by IMCA. Ultimately, overexpression of SLC7A11 restored the viability of DLD-1 and HCT116 cells inhibited by IMCA at 50 *μ*M. Taken together, our results provide a novel mechanism that IMCA induces cell death by ferroptosis through downregulating the expression of SLC7A11.

As a central energy metabolic switch, AMP-activated protein kinase (AMPK) exerts a paramount effect in cellular physiology and the pathological development of chronic diseases including cancer [51]. The activity of SLC7A11 is inhibited by AMPK phosphorylation through phosphorylating BECN1, which plays distinct roles in regulating cell ferroptosis [12, 32]. In addition, the activity of SLC7A11 is inhibited by the mTORC pharmacological inhibitor rapamycin, which counteracts the elevated expression of SLC7A11 induced by APR246 and the protective cellular responses, leading to cell death [34]. Consistent with these two mechanisms, DHA induces the lethal ROS accumulation and ferroptosis of leukemia cells through the AMPK/mTOR pathway [25]. The present study showed that IMCA at 50 *μ*M induced AMPK phosphorylation activation, mTOR dephosphorylation inhibition, and ultimately lethal ROS accumulation and ferroptosis in DLD-1 and HCT116 cells. Therefore, the SLC7A11 downregulation and ferroptosis induced by IMCA are related to the AMPK/mTOR pathway.

This study discovered a novel small-molecule compound (IMCA) for CRC treatment *in vitro* and *in vivo*, and elucidated that IMCA induces ferroptosis by downregulating SLC7A11 expression through the AMPK/mTOR pathway. These results provided a new therapeutic potential compound for CRC and new insights to induce ferroptosis.

## Figures and Tables

**Table 1:** The PCR primers.

CHAC1 reverse	5′-CCTGATGTCCACATGAGCACTCC-3′
CHAC1 forward	5′-ACCTTGAATACTTGCTGCGTCTGG-3′
PTGS2 reverse	5′-CCTGCTTGTCTGGAACAACTGCTC-3′
PTGS2 forward	5′-TGGTCTGGTGCCTGGTCTGATG-3′
GPX4 reverse	5′-GCAGCCGTTCTTGTCGATGAGG-3′
GPX4 forward	5′-CCGCTGTGGAAGTGGATGAAGATC-3′
GSS reverse	5′-AGCCTTCGGTCTTGGTCCAGAG-3′
GSS forward	5′-CCAGCGTGCCATAGAGAATGAGC-3′
SLC7A11 forward	5′-GGCTCCATGAACGGTGGTGTG-3′
SLC7A11 reverse	5′-GCTGGTAGAGGAGTGTGCTTGC-3′
*β*-Actin forward	5′-CATGTACGTTGCTATCCAGGC-3′
*β*-Actin reverse	5′-CTCCTTAATGTCACGCACGAT-3′

## Data Availability

All the data can be obtained from the corresponding authors.

## Supplementary Materials

Supplementary Materials Figure S1: IMCA did not significantly upregulate GPX4 expression. DLD-1 (a) and HCT116 (b) cells were treated with 50 *μ*M IMCA for 48 h, and the protein expression was assessed by Western blot. Figure S2: schematic illustration of IMCA for colorectal cancer therapy through inducing ferroptosis mediated by SLC7A11. The small molecule IMCA induces ferroptosis through downregulating the expression of SLC7A11, inhibiting the import of cystine, which resulted in glutathione (GSH) exhaustion. The downregulated expression of SLC7A11 is related to the phosphorylation of AMPK and dephosphorylation mediated by IMCA.
