# Corrigendum to “IMCA Induces Ferroptosis Mediated by SLC7A11 through the AMPK/mTOR Pathway in Colorectal Cancer”

**DOI:** 10.1155/2020/6901472

**Published:** 2020-10-27

**Authors:** Lei Zhang, Wen Liu, Fangyan Liu, Qun Wang, Mengjiao Song, Qi Yu, Kun Tang, Tieshan Teng, Dongdong Wu, Xijing Wang, Wuqi Han, Yanzhang Li

**Affiliations:** ^1^Institute of Biomedical Informatics, Bioinformatics Center, Laboratory for Nanomedicine, School of Basic Medical Sciences, Henan University, Kaifeng 475004, China; ^2^Department of Dermatology, Second People's Hospital of Zhengzhou, Zhengzhou 450006, China; ^3^Kaifeng Food and Drug Inspection Institute, Kaifeng 475004, China

In the article titled “IMCA Induces Ferroptosis Mediated by SLC7A11 through the AMPK/mTOR Pathway in Colorectal Cancer” [1], the authors have identified that the panels in Figure 1(h) were incorrectly duplicated due to an error in manuscript preparation. The corrected image is provided below.

## Figures and Tables

**Figure 1:**
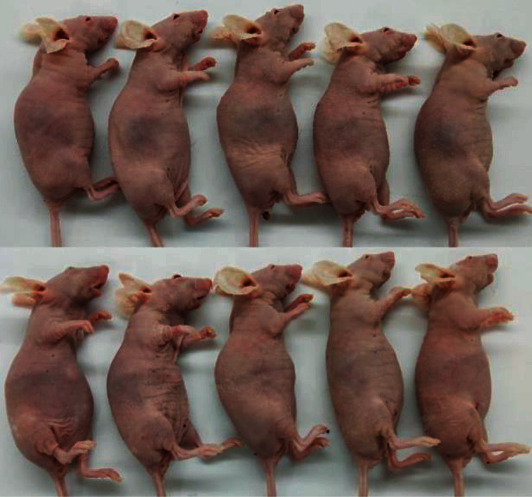


## References

[1] Zhang L., Liu W., Liu F. (2020). IMCA induces ferroptosis mediated by SLC7A11 through the AMPK/mTOR pathway in colorectal cancer. *Oxidative Medicine and Cellular Longevity*.

