# Supplementary material for: IMCA Induces Ferroptosis Mediated by SLC7A11 through the AMPK/mTOR Pathway in Colorectal Cancer
Source: Oxid Med Cell Longev. 2020 Apr 3;2020:1675613. doi: 10.1155/2020/1675613 (PMC7160732; doi:10.1155/2020/1675613)
Supplement: Supplementary Materials — Figure S1: IMCA did not significantly upregulate GPX4 expression. DLD-1 (a) and HCT116 (b) cells were treated with 50 μM IMCA for 48 h, and the protein expression was assessed by Western blot. Figure S2: schematic illustration of IMCA for colorectal cancer therapy through inducing ferroptosis mediated by SLC7A11. The small molecule IMCA induces ferroptosis through downregulating the expression of SLC7A11, inhibiting the import of cystine, which resulted in glutathione (GSH) exhaustion. The downregulated expression of SLC7A11 is related to the phosphorylation of AMPK and dephosphorylation mediated by IMCA. [file 1675613.f1.pdf]

Supplementary materials

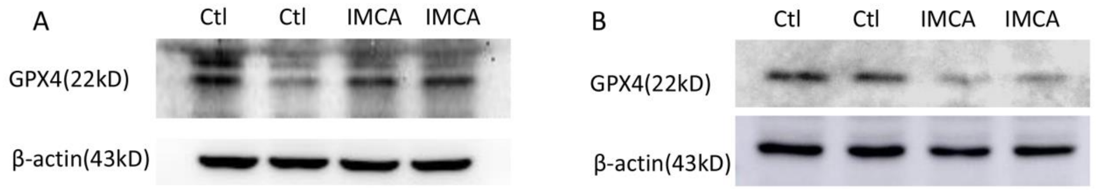

Figure S1. IMCA did not significantly up-regulate GPX4 expression. DLD-1 (A) and HCT116 (B) cells were treated with 50 μM IMCA for 48h, and the protein expression was assessed by western blot.

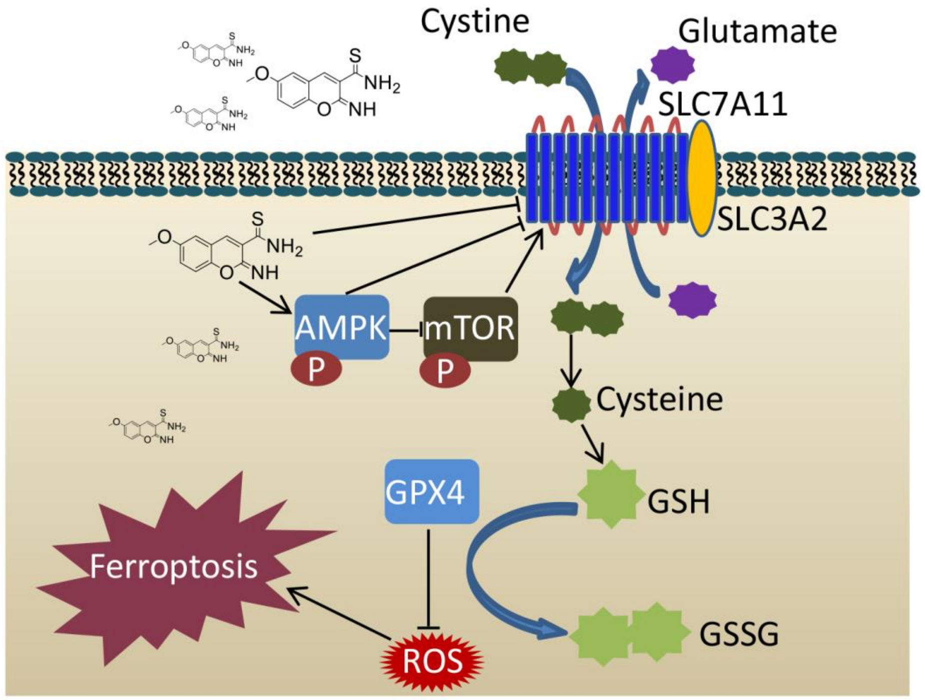

Figure S2. Schematic Illustration of IMCA for colorectal cancer therapy through inducing ferroptosis mediated by SLC7A11. The small molecule IMCA induces ferroptosis through down-regulating the expression of SLC7A11, inhibiting the import of cystine, which resulted in glutathione (GSH) exhaustion. The down-regulating the expression of SLC7A11 is related to the phosphorylation of AMPK and dephosphorylation mediated by IMCA.
